# Genetic prevalence of repeat expansion and other rare neurological disorders in the Quebec population

**DOI:** 10.3389/fgene.2026.1964104

**Published:** 2026-10-21

**Authors:** Annelie De Mori, Marjorie Labrecque, Valérie Triassi, Sebastien Audet, Justin Pelletier, Abbe Lai, Roberta La Piana, Antoine Duquette, Claude Bhérer, Martine Tetreault

**Affiliations:** 1 Department of Biochemistry and Molecular Medicine, Université de Montréal, Montreal, QC, Canada; 2 Centre de recherche du Centre Hospitalier de l’Université de Montréal (CRCHUM), Université de Montréal, Montreal, QC, Canada; 3 Department of Neurosciences, Université de Montréal, Montreal, QC, Canada; 4 Department of Human Genetics, McGill CERC Program in Genomic Medicine, Victor Phillip Dahdaleh Institute of Genomic Medicine at McGill University, Montreal, QC, Canada; 5 Department of Neurology and Neurosurgery, Montreal Neurological Institute-Hospital, McGill University, Montreal, QC, Canada; 6 Neurology Service, Department of Medicine, André-Barbeau Movement Disorders Unit, Centre Hospitalier de l’Université de Montréal (CHUM), Montreal, QC, Canada; 7 Genetic Service, Department of Medicine, Centre Hospitalier de l’Université de Montréal (CHUM), Montreal, QC, Canada

**Keywords:** carrier frequency, founder effect, prevalence, quebec, rare neurological disorders, repeat expansions

## Abstract

**Background:**

Repeat expansion disorders (REDs) are a class of genetic disease, most of which cause neurological or neurodevelopmental phenotypes. Recent improvements in repetitive sequence detection suggest that REDs are collectively more common than previous estimates. Several REDs occur in Quebec at exceptional frequencies, due to founder effects which have shaped the province’s genetic landscape. Because these disorders are clinically heterogeneous and difficult to diagnose, their true prevalence in the province remains largely unknown.

**Methods:**

By using whole-genome, exome, and RNA-sequencing data from CARTaGENE (CaG), a prospective Quebec population-based cohort, we characterized 13 RED loci as well as two genes associated to non-repeat neurological disorders, autosomal recessive cerebellar ataxia type 1 (ARCA1) and hereditary spastic paraplegia 11 (HSP11).

**Results:**

We estimated carrier and expansion frequencies of premutation and full expansion alleles, finding that FXN, DMPK and FGF14 exhibited the highest values in Quebec. Assuming Hardy-Weinberg equilibrium, we calculated a genetic prevalence of 55.28 per 100,000 for SCA27B, a disorder for which prevalence estimates are lacking. Several additional REDs’ genetic prevalences were estimated up to 6.5 times higher than previously reported figures, indicating possible underdiagnosis or incomplete penetrance. Other RED genetic prevalences in CaG remained comparable to reported values, indicating that the founder effect acts on specific loci rather than across all repeat expansions. These findings highlight the REDs for which clinical resources should be prioritized in the province. Candidate founder variants associated with ARCA1 and HSP11 were identified by an enrichment analysis comparing CaG allele frequencies to the gnomAD Non-Finnish European cohort, and we report province-wide genetic prevalence estimates for both conditions.

**Conclusion:**

Using CaG’s sequencing data, we established a genetic profile of rare neurological disorders in Quebec. These results provide valuable information for the development of genetic screening and diagnosis strategies for rare neurological diseases.

## 1 Introduction

Understanding what proportion of individuals in the general population carry a disease-causing variant is one of the central questions in medical genetics. Accurate estimates of disease prevalence and carrier frequency (CF) are essential for planning genetic screening programs, improving diagnostic pathways, and guiding public health decisions. For rare genetic diseases in particular, these guiding values have been hard to define, as prevalence estimates have mostly relied on clinical case registries, which are often not available and can be limited by diagnostic delays, misclassification, and the omission of mildly affected or presymptomatic individuals. Population-based prospective cohorts offer an alternative by recruiting individuals from the general population, making it possible to calculate allele frequencies, carrier rates, and genetic prevalences across a representative sample of the population (Labrecque et al., 2021; Ibañez et al., 2024; Pounraja et al., 2026). The increasing accessibility of genetic sequencing has made this approach feasible even for rare disorders, allowing carrier frequencies to be estimated directly from sequencing data without the biases of clinical datasets (Ibañez et al., 2024).

Prospective cohorts are also valuable for studying populations with unique histories, such as Quebec’s French-Canadian population. Quebec’s genetic landscape was largely shaped by a founder effect that began when approximately 8,500 French settlers arrived in the region between 1608 and 1759 (Laberge et al., 2005). Three distinct migration waves created a triple founder effect, impacting the Saint-Lawrence Valley, Charlevoix, and Saguenay-Lac-Saint-Jean (SLSJ) (Supplementary Figure S1; Cruz Marino et al., 2023). These founder effects influenced the geographic distribution of genetic diseases in Quebec and resulted in lower genetic variation compared to other populations. Consequently, certain deleterious or pathogenic variants are much more common in French Canadians than in other populations (Laberge et al., 2005). These conditions follow the typical patterns of founder diseases, such as a higher prevalence in the population due to the lower genetic variation. For example, Autosomal Recessive Spastic Ataxia of Charlevoix-Saguenay (ARSACS) is notable for its higher prevalence in Charlevoix and SLSJ, where approximately one in 22 individuals are carriers of ARSACS-associated variants (De Braekeleer et al., 1993).

Some repeat expansion disorders (REDs) are among the genetic disorders that are relatively frequent in the French-Canadian population. Expansions of short tandem repeats (STR) occur when the number of repeated units of a specific DNA motif increases beyond a locus-specific threshold associated with pathogenicity (Paulson, 2018). The expansion size can increase across generations due to instability during meiosis, a phenomenon known as anticipation, which may contribute to cases with no apparent family history (Paulson, 2018). Repeat lengths can be classified as normal, premutation/intermediate or pathogenic in accordance to gene-specific thresholds. Premutation alleles are highly prone to expand into fully penetrant pathogenic expansions during transmission, and in some cases have been reported to cause reduced penetrance clinical manifestations of the disorder. There are around 60 REDs characterized as of now, most associated with neurological or neurodevelopmental diseases (Chen et al., 2025). Recent estimates suggest that REDs are collectively more common than once believed, with a combined allele frequency affecting one in 283 individuals (Chen et al., 2025). In the French-Canadian population, REDs have reached exceptional frequencies due to founder effects, making Quebec a critical landscape for their study. This is exemplified by Myotonic Dystrophy Type 1 (DM1) and Oculopharyngeal Muscular Dystrophy (OPMD), both of which show some of the highest prevalence rates globally within the province (Mathieu and Prévost, 2012; Bouchard, 1997; Bouchard et al., 1997). The recent discovery of an expansion in the *FGF14* gene associated with Spinocerebellar Ataxia 27B (SCA27B) highlights the ongoing identification of new pathogenic variants in this population (Pellerin et al., 2023). REDs exhibit a broad range of neurological presentations, and symptoms for a given disease can vary depending on age at onset, repeat structure or the presence of interruptions, making these disorders challenging to diagnose. Because these diseases are rare and their symptoms often overlap, many patients remain undiagnosed or misdiagnosed, leaving the true prevalence of these repeat expansion diseases in the province largely unknown.

Beyond repeat expansion disorders, Quebec’s founder history has also contributed to the enrichment of specific variants in other rare neurological conditions, such as autosomal recessive cerebellar ataxia type 1 (ARCA1) and hereditary spastic paraplegia type 11 (HSP11). ARCA1 is an autosomal recessive neurodegenerative disorder characterized by a slowly progressive cerebellar ataxia associated with dysarthria (Dupré et al., 2007). It was first described in a French-Canadian cohort and is caused by variants in the *SYNE1* gene. HSP11 is a neurodegenerative disorder characterized by progressive muscle stiffness and lower-limb paralysis, caused by variants in the *SPG11* gene (Kang et al., 2023). *SPG11* variants are also associated with progressive cognitive deficits (Chrestian et al., 2017). ARCA1 and HSP11 variants have been reported at high frequency in the Beauce region compared to other parts of Quebec, and HSP11 was found to be the most frequent autosomal recessive hereditary spastic paraplegia (HSP) in an observational study of patients from Alberta, Ontario, and Quebec (Gagnon et al., 2025; Chrestian et al., 2017). However, there are currently no published prevalence estimates for ARCA1 or HSP11 across the province.

To address these gaps, this study uses CARTaGENE (CaG), a unique prospective cohort of Quebec residents (Awadalla et al., 2013; McClelland et al., 2026). The CaG cohort is the largest population-based cohort in Quebec, composed of 43,032 participants aged 40–69 years, recruited from multiple metropolitan areas of the province (McClelland et al., 2026). Launched in 2007, this resource was developed to support genetic and health research across the province’s population. Our goal is to establish a genetic profile of rare and founder variants, and to measure the prevalence of neurological diseases in Quebec using sequencing data from CaG. Estimating carrier frequencies and genetic prevalence is essential for improving genetic diagnosis, evaluating the risk of rare neurological disorders, and designing genetic screening strategies.

## 2 Materials and methods

### 2.1 Sequencing datasets

From the CaG cohort, 2,184 short-read whole genome sequencing (WGS), 911 RNA-sequencing (RNA-seq) and 198 exome-sequencing (exome-seq) samples were generated and used in our analyses. Across these 3,293 datasets, 104 individuals were present in at least two of the three groups, leaving sequencing data on 3,189 distinct individuals. At the time of recruitment, no participant in the CaG cohort had received a diagnosis of a rare neurological disease. The WGS participants selected by CaG were unrelated and had all four grandparents born in the same country, most often Canada and, to a lesser extent, Haiti or Morocco. Within the CaG cohort, 61% of all participants and 80.1% of the WGS subset were Quebec residents of French-Canadian ancestry (McClelland et al., 2026).

### 2.2 Repeat expansion genotyping

ExpansionHunter (EH, v5.0.0) was used to estimate repeat sizes of RED loci from the WGS data, aligned to the GRCh38 reference genome (Dolzhenko et al., 2017; Dolzhenko et al., 2019). Based on a predefined variant catalog specifying the genomic location and structure of each STR, EH constructs a sequence graph for each locus and realigns reads to estimate the repeat genotype. The standard EH variant catalog was modified to add the *FGF14* repeat expansion locus including off-target regions from STRipy (Halman et al., 2022; Mohren et al., 2024). Repeat sizes were classified as premutations or pathogenic according to locus-specific thresholds (Table 1). ExpansionHunter Denovo (EHdn, v0.9.0) was additionally used to detect the expansion motifs in *RFC1*, which determine the pathogenicity of the expansion (Dolzhenko et al., 2020). In contrast to EH, EHdn uses a catalog-free method to identify novel expansions and can detect nucleotide composition changes within the repeats.

**TABLE 1:** RED-loci included in the study, including repeat-size thresholds.

Disease	Locus	Motif	Premutation/Reduced penetrance repeat size	Pathogenic repeat size
Spinal and bulbar muscular atrophy (SBMA)	*AR*	CAG	35–37	38+
Dentatorubral-pallidoluysian atrophy (DRPLA)	*ATN1*	CAG	35–47	48+
Spinocerebellar ataxia 1 (SCA1)	*ATXN1*	CAG	39–43	44+
Spinocerebellar ataxia 2 (SCA2)	*ATXN2*	CAG	33–34	35+
Spinocerebellar ataxia 3 (SCA3)	*ATXN3*	CAG	45–59	60+
Spinocerebellar ataxia 7 (SCA7)	*ATXN7*	CAG	34–36	37
Amyotrophic lateral sclerosis (ALS)/Frontotemporal dementia (FTD)	*C9orf72*	GGGGCC	-	30+
Spinocerebellar ataxia 6 (SCA6)	*CACNA1A*	CAG	19	20+
Myotonic dystrophy type 1 (DM1)	*DMPK*	CTG	35–49	50+
Spinocerebellar ataxia 27B (SCA27B)	*FGF14*	GAA	250–300	301+
Fragile X syndrome (FXS)	*FMR1*	CGG	55–200	201+
Fragile X-associated tremor/ataxia syndrome (FXTAS)	*FMR1*	CGG	-	55–200
Friedreich’s ataxia (FRDA)	*FXN*	GAA	45–65	66+
Huntington’s disease (HD)	*HTT*	CAG	36–39	40+
Huntington’s disease-like 2 (HDL2)	*JPH3*	CAG	29–39	40+
Neuronal intranuclear inclusion disease (NIID)	*NOTCH2NL*	GGC	40–59	60+
Oculopharyngeal muscular dystrophy (OPMD)	*PABPN1*	GCG	-	11–18
Spinocerebellar ataxia 12 (SCA12)	*PPP2R2B*	CAG	-	51+
Cerebellar ataxia, neuropathy and vestibular areflexia syndrome (CANVAS)	*RFC1*	AAGGG/ACAGG	-	250+
Spinocerebellar ataxia 17 (SCA17)	*TBP*	CAG/CAA	41–48	49+

### 2.3 Computation of carrier frequencies, expansion frequencies and genetic prevalence

The carrier and expansion frequencies of premutation and pathogenic alleles were estimated for each RED locus present in our dataset. In addition, genetic prevalence was estimated for loci with expansion alleles and only included the number of alleles with repeats larger than the pathogenic threshold. Affected births were calculated according to the most recent available birthrate in Quebec of 78,200 in 2025 (Institut de la statistique du Québec, 2026).

#### 2.3.1 Carrier and expansion frequency estimates (1 in x)

Frequencies were estimated as described previously by Ibañez et al. and were calculated separately for full-expansion and premutation alleles at each locus (Ibañez et al., 2024). For a given STR type, the point estimate of the disease allele frequency was calculated as p = e/n, where e is the number of expanded alleles identified and n is the total number of unrelated genomes analyzed, except for X-linked loci, where n was the number of male or female participants. For autosomal recessive loci, premutation alleles at any locus, and pathogenic alleles in females at X-linked recessive loci, this value is reported as a carrier frequency. For pathogenic-range expansions at autosomal dominant loci and in males at X-linked recessive loci, this value is instead reported as an expansion frequency. Both are reported as “1 in x” by taking round(1/p). Ninety-five percent confidence intervals were derived using the Wilson score interval (z = 1.96):

Confidence interval (CI) = $\frac{p+\frac{z^{2}}{2n}\;\pm \;z\times \sqrt{\frac{p\times q}{n}+\frac{z^{2}}{4n^{2}}}}{1+\frac{z^{2}}{n}}$ , where q = 1 – p. The upper and lower bounds of the frequency interval were obtained with round(1/CI_max) and round(1/CI_min), respectively.

#### 2.3.2 Genetic prevalence estimate (x in 100,000)

Genetic prevalence was estimated, as described by Labrecque et al., assuming Hardy-Weinberg equilibrium p^2^ + 2pq + q^2^ = 1, where q is the frequency of disease-causing alleles (Labrecque et al., 2021). This prevalence represents the expected proportion of individuals with a disease-associated genotype, assuming full penetrance. For each locus, the allele frequency was calculated as q = ac/an, where ac is the number of disease-associated alleles and an is the total number of alleles observed.

##### 2.3.2.1 Autosomal recessive diseases

For autosomal recessive disorders, the proportion of individuals expected to develop the disease is given by the frequency of homozygotes, q^2^. The expected number of affected births was calculated as q^2^ x 78,200, and genetic prevalence was expressed as q^2^ x 100,000 cases per 100,000 individuals.

##### 2.3.2.2 Autosomal dominant diseases

For autosomal dominant disorders, affected individuals are predominantly heterozygous, so the proportion expected to develop the disease is 2q: 2q(1–q), which simplifies to approximately 2q when q is small. Because this estimate tends to overestimate prevalence, we calculated 95% confidence intervals for q using a Bayesian binomial approach, implemented with the binom.bayes() function from the binom R package, and took the lower bound of the interval as our prevalence estimate (Dorai-Raj, 2022). The expected number of affected births was calculated as 2q x 78,200, and genetic prevalence was expressed as 2q x 100,000 cases per 100,000 individuals.

##### 2.3.2.3 X-linked diseases

Genetic prevalence of X-linked diseases was estimated separately for males and females. In males, the disease frequency is equal to the allele frequency q. As above, binomial confidence intervals were used to limit overestimation. Affected male births were calculated using the 2025 Quebec male birth count of 40,227, and male genetic prevalence was estimated as q x 100,000 cases per 100,000 individuals (Institut de la statistique du Québec, 2026). In females, genetic prevalence was estimated using the autosomal recessive or autosomal dominant approach described above, depending on whether the disorder was X-linked recessive or dominant. Affected female births were calculated using the 2025 Quebec female birth count of 37,973 (Institut de la statistique du Québec, 2026).

### 2.4 Identification of variants in *SYNE1* and *SPG11*

Genomic variants were identified and characterized in the *SYNE1* and *SPG11* genes, causing ARCA1 and HSP11 respectively, by analysing the WGS, RNA-seq and exome-seq samples from CaG.

The pipeline for identification and classification of ARCA1 and HSP11 variants in RNA-seq and exome-seq data was used as described by Touma et al. (2021). The RNA and exome sequences were aligned to the GRCh37 human reference genome. RNA-seq data were aligned with HISAT2 and processed with VarDict, while exome-seq data were aligned with BWA and called using GATK. All variants were annotated using ANNOVAR and internal scripts to integrate pathogenicity (CADD, SIFT, PolyPhen-2), conservation (phastCons, GERP), and population frequency (gnomAD) metrics. To isolate rare variants, we filtered them based on an allele frequency (AF) < 1%, CADD >15, and high pathogenicity and conservation thresholds (inverted SIFT >0.75, PolyPhen-2 > 0.75, GERP >5, phastCons >500). Finally, *SYNE1* and *SPG11* pathogenic and likely pathogenic variants were selected according to ACMG guidelines, or by their classification in ClinVar and the literature. Candidate variants were confirmed by visual inspection with IGV, and only variants supported by a minimum of 10 reads, with at least 20% of reads carrying the variant allele, were retained.

To identify variants from the more recent WGS data, another pipeline was implemented using the GRCh38 human reference genome. To ensure cohort-wide consistency, CaG performed the initial alignment to provide standardized data for all groups (McClelland et al., 2026). Custom scripts (available upon request) were implemented to extract and process the regions of interest by annotating with ANNOVAR and SnpEff, as well as adding allelic frequencies, pathogenicity and conservation scores. The same variant prioritization process as the RNA and exome-sequencing data was used for WGS.

Allele frequencies of each variant within the CaG cohort were calculated by dividing the number of alternate alleles by the total number of alleles at each locus. To identify variants enriched in the CaG participants, these frequencies were compared against the gnomAD Non-Finnish European (NFE) cohort (v4.1.0). Statistical significance and odds ratios were determined using a one-sided Fisher’s exact test, implemented in R version 4.3.3, to identify variants with significantly higher enrichment in the CaG cohort.

## 3 Results

### 3.1 RED variant frequency

To estimate the number of individuals carrying premutation or full-expansion alleles, we selected RED genes that are frequent in the Quebec population, reported in multiple French-Canadian families, recently discovered expansions, or for which premutation and full-expansion alleles can be accurately distinguished using WGS (Ibañez et al., 2024). In total, we included 19 RED loci in our analysis, five of which have suspected higher frequencies in the French-Canadian population according to the literature: SCA2 (*ATXN2*), DM1 (*DMPK*), SCA27B (*FGF14*), FRDA (*FXN*), and OPMD (*PABPN1*) (Alshimemeri et al., 2023; Daoud et al., 2012; Yotova et al., 2005; Pellerin et al., 2023; Barbeau et al., 1984; Bouchard, 1997). ALS/FTD (*C9orf72*) and SCA27B have additionally been reported in multiple French-Canadian families, and CANVAS (*RFC1*) is a more recently characterized expansion (Daoud et al., 2012; Davies et al., 2022). A French-Canadian cohort showed a high female carrier frequency of *FMR1* premutations causing FXTAS (Sherman, 1995; Rousseau et al., 1995). Most of these loci follow autosomal dominant inheritance, except FRDA and CANVAS which are autosomal recessive, SBMA (*AR*) which is X-linked recessive, and FXS (*FMR1*) which is X-linked dominant. Based on literature review, we defined the premutation and pathogenic thresholds used to identify variants (Table 1). To determine which RED variants were the most frequent in our cohort, we summed the number of premutation and expanded alleles together for each RED locus. Out of the 19 genes analysed, 13 had disease-associated premutations or expansions in the CaG cohort. The largest number of premutation and pathogenic expansions was found in *FXN*, followed by *DMPK*, *FGF14*, *FMR1*, *TBP* and *AR* (Figure 1). Expansions were also identified in *C9orf72*, *HTT*, *JPH3*, *ATN1*, *ATXN1*, *ATXN2* and *ATXN7* (Figure 1). The remaining six loci had no identified premutations or expansions: *ATXN3, CACNA1A, NOTCH2NL, PABPN1, PPP2R2B, RFC1*. Among the 13 genes with variants, six harboured pathogenic expansions, most of which are associated with diseases expected to be frequent in the French-Canadian population.

**FIGURE 1:**
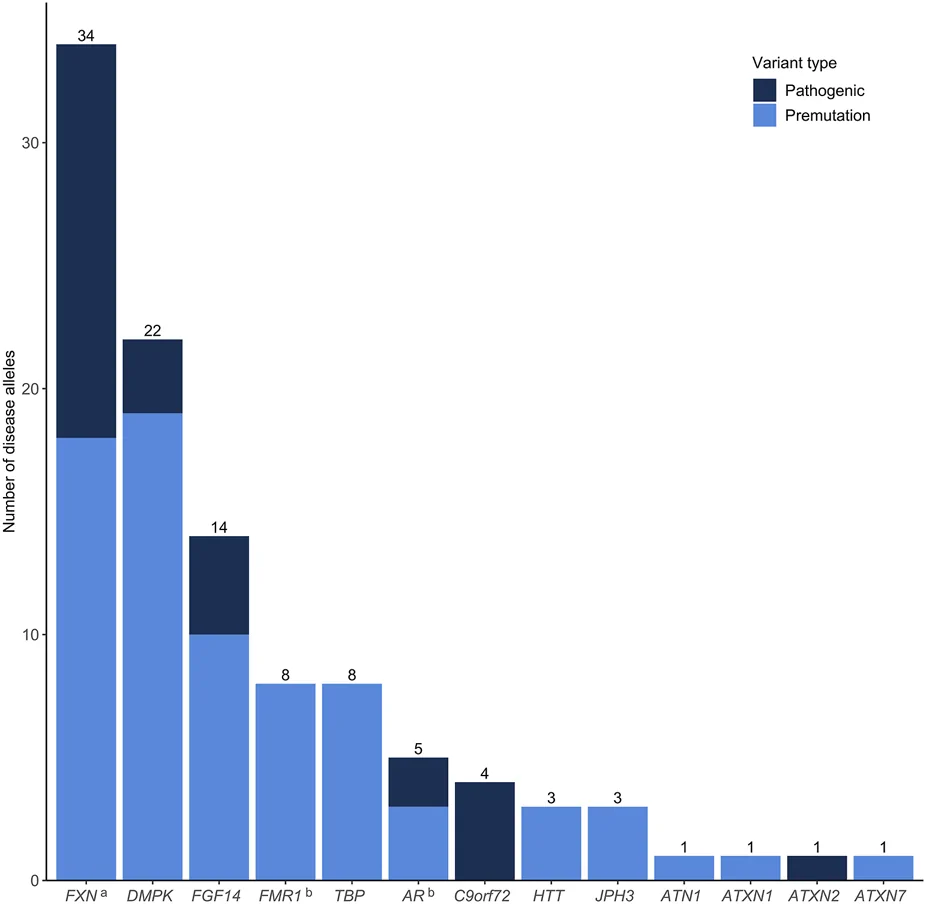
Number of premutation and pathogenic expansions identified in each gene. Each bar shows the total number of STRs identified in each gene, divided into premutation alleles (light blue) and pathogenic alleles (dark blue). Autosomal recessive and X-linked loci are indicated by a and b respectively. All other loci are autosomal dominant.

We estimated the number of individuals carrying premutation or full-expansion alleles to determine how frequent these variants are in the Quebec population. In the CaG cohort, *FXN* had the most common pathogenic expansion carrier frequency, at one in 136 (Figure 2). *FXN* premutation carriers were even more common, at one in 121. This trend was observed across all genes where both variant types were identified, consistent with the expectation that alleles below the full-penetrance threshold are more common in the general population.

**FIGURE 2:**
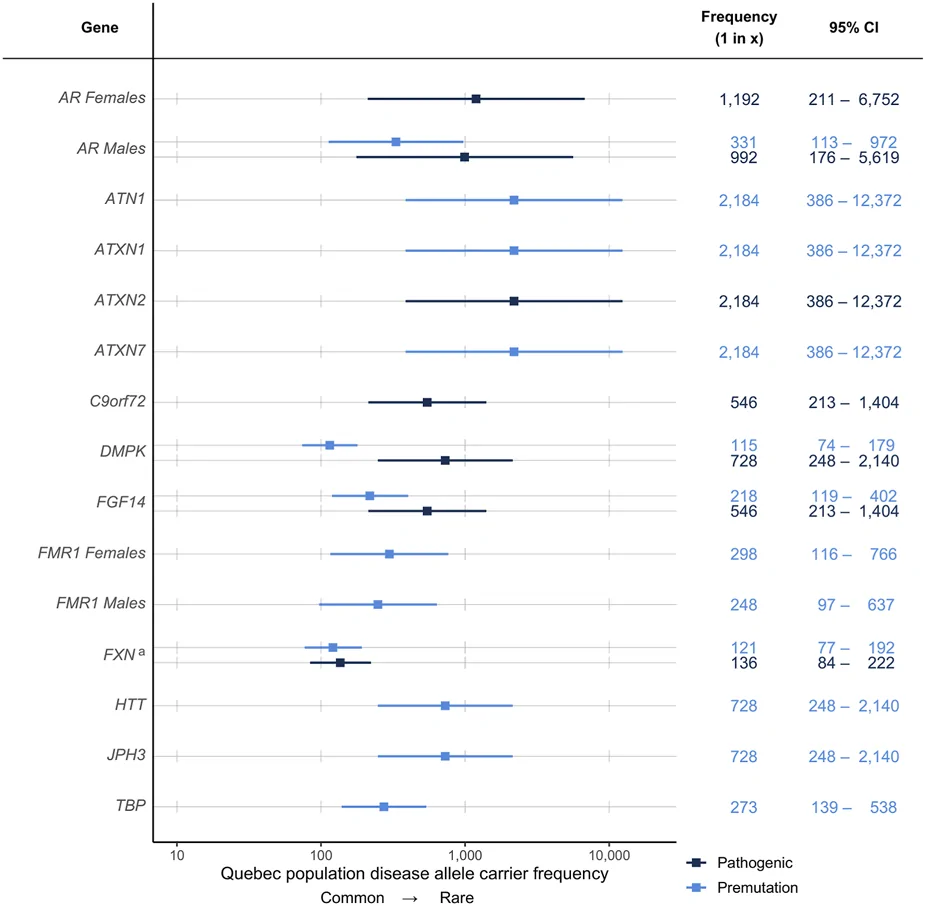
Forest plot of disease allele carrier and expansion frequencies in the CaG cohort. The squares show the estimated carrier or expansion frequency for each locus, and the bars show the corresponding 95% CI values. The light blue squares indicate premutation or reduced-penetrance allele carrier frequencies, and dark blue squares indicate pathogenic-range allele expansion frequencies. Autosomal recessive loci are marked with a, and X-linked loci are shown with separate male and female CFs.

Among the dominant expansion disorders, *FGF14* and *C9orf72* showed the highest pathogenic expansion frequencies, both at one in 546, followed by *DMPK* at one in 728 (Figure 2). Unlike the other loci in this group, *C9orf72* does not have a defined premutation range (Table 1), and all identified alleles were therefore classified as pathogenic expansions. For *DMPK* and *FGF14*, premutation CFs were elevated relative to pathogenic expansion frequencies, most notably one in 115 for *DMPK,* the highest estimated frequency regardless of variant type in this cohort, and one in 218 for *FGF14*. *ATXN2* harboured one pathogenic allele, yielding an expansion frequency of one in 2,184. Only premutation alleles were observed at the remaining dominant expansion loci. *TBP* yielded a premutation CF of one in 273, followed by *HTT* and *JPH3*, both at one in 728. A single premutation allele was observed for each of *ATN1*, *ATXN1* and *ATXN7*, resulting in a CF of one in 2,184.

To assess the regional origin of the *DMPK* and *FXN* carriers, a principal component analysis (PCA) was performed using region-labelled CaG European descent participants (McClelland et al., 2026) and carriers (Supplementary Figure S2). Genetic affinity was quantified by k-nearest-neighbors classification on the balanced CaG PCA. For *DMPK*, no carrier could be confidently assigned a region of origin. Using CaG’s previously described Identity-By-Descent (IBD) cluster analysis, two *DMPK* premutation carriers and one full-expansion carrier grouped within a Saguenay-enriched cluster (McClelland et al., 2026). This cluster includes individuals recruited across multiple regions of Quebec and likely reflects shared ancestry from early founder populations that contributed to the settlement of Saguenay as well as other regions of the province, rather than ancestry specific to the Saguenay region. For *FXN*, apart from one carrier assigned to Trois-Rivières, no carrier could be assigned a region of origin, and four carriers grouped with the same Saguenay-enriched IBD cluster as carriers of *DMPK*. These distributions were consistent across the balanced and unbalanced PCAs for both genes.

The two X-linked loci, *AR* and *FMR1*, required sex-stratified analysis, with frequencies calculated separately among the 992 male and 1,192 female participants. No female premutation carriers of *AR* were identified; male premutation carriers were estimated at one in 331. Pathogenic *AR* alleles were identified in one male and one female participant, corresponding to an expansion frequency of one in 992 in males and a carrier frequency of one in 1,192 in females (Figure 2). For *FMR1*, no pathogenic alleles were identified in any participant. Premutation alleles were equally distributed between sexes, yielding CFs of one in 248 in males and one in 298 in females.

Taken together, this data highlights DM1, FRDA, and SCA27B as REDs with a high frequency in the general Quebec population. Of the five prevalent REDs examined (*FXN*, *DMPK*, *FGF14*, *PABPN1*, and *ATXN2*) four harboured disease-causing alleles in the CaG cohort, three of which (*DMPK*, *FXN*, *FGF14*) showed a higher frequency than the remaining loci analyzed.

### 3.2 Prevalence estimation in the CaG cohort

To determine whether these repeat expansion diseases are enriched within our cohort, we estimated their genetic prevalence in CaG and compared these estimates to the global prevalences reported in the literature (Table 2; Figure 3). For REDs with X-linked or autosomal dominant inheritance, we calculated binomial confidence intervals using Bayesian inference and used the lower interval to offset overestimation due to sample size. Only REDs with identified pathogenic expansions were included in prevalence calculations.

**TABLE 2:** Estimated genetic prevalences in the CaG cohort and reported prevalences in the literature.

Disease	Prevalence in CaG (per 100,000)	Global prevalence (per 100,000)	Quebec prevalence (per 100,000)
SBMA female	0.018	-	-
SBMA male	4.29	3.33	-
FRDA	1.34	0.5	0.18^a^
SCA2	1.94	1–2	1.06^a^
DM1	32.64	5	158^b^
ALS/FTD	55.28	5–12/9.17	-
SCA27B	55.28	-	-

^a^ Minimum prevalence in Eastern Quebec ^b^ Prevalence in the SLSJ region.

**FIGURE 3:**
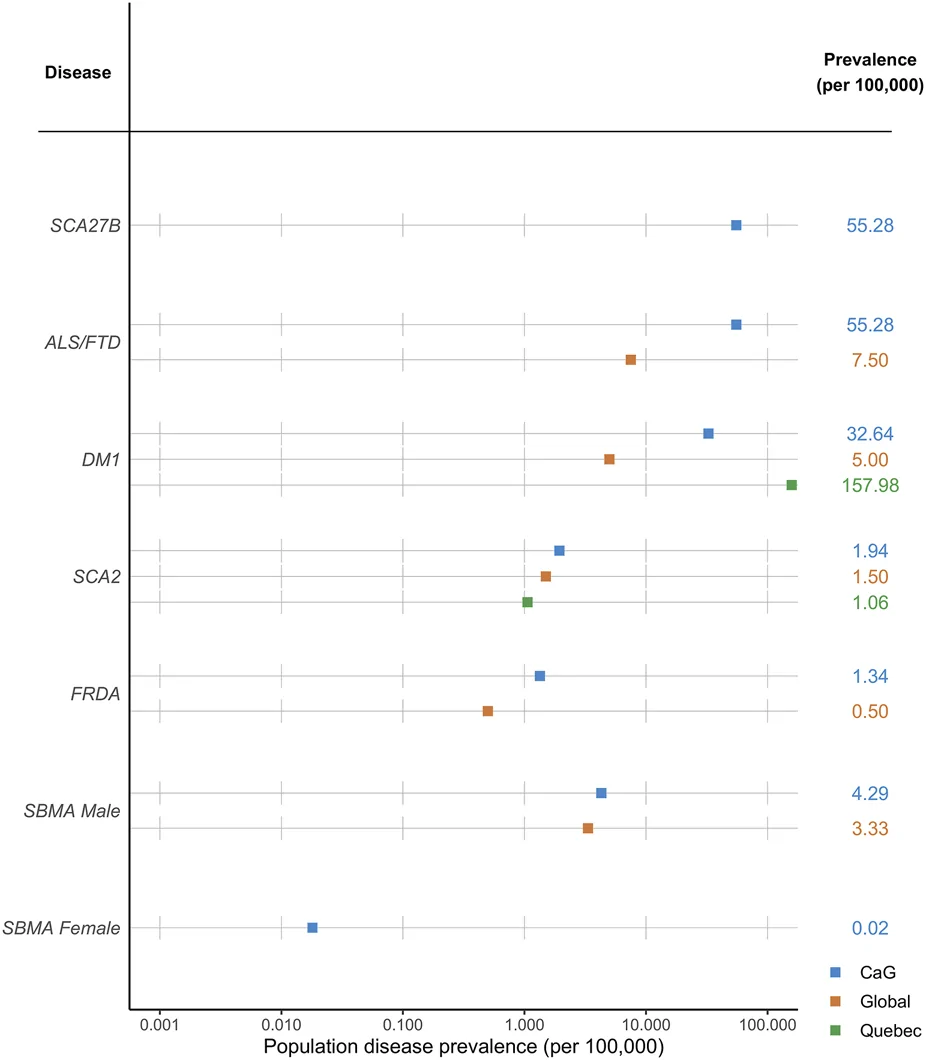
Comparison of estimated genetic prevalences in the CaG cohort and reported prevalences in the literature. The squares show the genetic prevalence for each gene harbouring pathogenic expansions in the CaG cohort. The blue, orange and green squares show the estimated genetic prevalence in this study, the reported global prevalence and the reported Quebec prevalence respectively. Global prevalences reported in ranges are represented as the mid-point of the range. For autosomal dominant and X-linked loci, the CaG square represents the lower bound of the 95% CI, used as a conservative estimate to limit overestimation. Genetic prevalences for X-linked loci are separated by sex.

In the CaG cohort, we estimated a genetic prevalence of 55.28 per 100,000 for both SCA27B and ALS/FTD (Table 2). This corresponds to approximately 43 expected affected births per year in Quebec for each disorder, based on the 78,200 births recorded in 2025. As SCA27B is a recently described RED, its worldwide prevalence has not yet been estimated, but it is the most common genetic cause of adult-onset ataxia in the French-Canadian population (Pellerin et al., 2024). Our estimated prevalence for ALS/FTD is almost 5 times higher than the reported prevalence of ALS (5–12 per 100,000 (Gossye et al., 1993)) and 6 times higher than FTD’s (9.17 per 100,000 (Urso et al., 2025)) (Table 2; Figure 3).

For DM1, we estimated a genetic prevalence of 32.64 per 100,000, 6.5 times higher than the global prevalence (5 per 100,000 (Liao et al., 2022)), although 4.8 times lower than the reported prevalence for the SLSJ region (158 per 100,000 (Mathieu and Prévost, 2012)). This is equivalent to approximately 26 expected affected DM1 births per year in Quebec. For SCA2, our estimated genetic prevalence of 1.94 per 100,000 falls at the upper end of the reported range of 1–2 per 100,000 and is comparable to the current minimum prevalence in Eastern Quebec of 1.06 per 100,000 (Haj Salem et al., 2021; Orphanet, 2026). This corresponds to approximately 1.5 expected affected births per year.

As an autosomal recessive disease, FRDA has a higher carrier frequency than most other REDs. Its estimated genetic prevalence of 1.34 per 100,000 is 2.7 times higher than the reported prevalence of 0.5 per 100,000, and is equivalent to 1.05 affected births per year in Quebec (Buesch and Zhang, 2022). SBMA, an X-linked recessive disease, required sex-stratified prevalence estimates. The male SBMA genetic prevalence in CaG was 1.3 times higher than the reported male prevalence (4.29 per 100,000 vs. 3.33 per 100,000 (Zanovello et al., 2023)), while the female genetic prevalence was estimated at 0.018 per 100,000. As very few women develop SBMA, no reported prevalence is available for comparison. These values correspond to 1.73 affected male births per year, and 0.0068 affected female births.

Overall, these estimates could indicate potential founder effects for REDs with an elevated genetic prevalence, or a higher frequency of these variants in Quebec than previously thought. The observed genetic prevalences may also be influenced by population-wide underdiagnosis or incomplete penetrance among individuals carrying repeats above the pathogenic threshold.

### 3.3 Enrichment of ARCA1 and HSP11 variants in CaG

Since variants in both ARCA1 and HSP11 have been reported at high frequency in the Beauce region, we sought to identify which variants for these diseases are present in the Quebec population and to estimate their carrier frequency and genetic prevalence. We found seven variants classified as pathogenic in *SYNE1*. The most frequent in CaG was c.15918–12A>G, for which we found ten alleles, including one homozygous carrier. We identified seven alleles carrying c.16390–2A>G and six carrying c.8695A>T, as well as a single c.17816_17820del allele. These four variants have all been previously reported among individuals of French-Canadian descent (Dupré et al., 2007). We also found carriers of three additional pathogenic ARCA1 variants: c.14500C>T, c.14211dup and c.1903dup.

For *SPG11*, we identified three variants: c.1478_1482del, c.6598A>T and c.6100C>T. c.1478_1482del was the most common variant in the CaG cohort, while c.6598A>T had previously been found at higher frequency in the Beauce region (Gagnon et al., 2025).

A total of 3,189 distinct individuals were included across the WGS, RNA-seq, and exome-seq samples, giving 6,378 alleles. For *SYNE1*, all allele frequencies calculated in CaG were higher than the corresponding AFs in NFE from the gnomAD database (v4.1.0). All variants except c.17816_17820del were significantly enriched in CaG, with c.14211dup the most significantly enriched (Table 3; Figure 4).

**TABLE 3:** Pathogenic variants in the S*YNE1* gene.

Variant	Allele count	CaG AF	CaG AF CI	gnomAD NFE AF	P-value	Odds ratio
c.8695A>T	7	1.10e-03	5.91e-04–2.59e-03	1.441e-05	1.16e-11	91.66
c.14500C>T	1	1.57e-04	2.04e-05–8.82e-04	3.390e-06	0.022	55.63
c.15918–12A>G	10	1.57e-03	9.70e-04–3.34e-03	3.898e-05	9.53e-14	48.36
c.16390–2A>G	4	6.27e-04	2.55e-04–1.79e-03	5.932e-06	1.29e-07	126.82
c.17816_17820del	1	1.57e-04	2.04e-05–8.82e-04	9.322e-06	0.052*	20.23
c.1903dup	5	7.84e-04	3.60e-04–2.07e-03	2.543e-06	9.94e-11	373.93
c.14211dup	12	1.88e-03	1.24e-03–3.83e-03	8.474e-07	2.20e-16	2870.19

*Non-significant p-values (>0.05).

**FIGURE 4:**
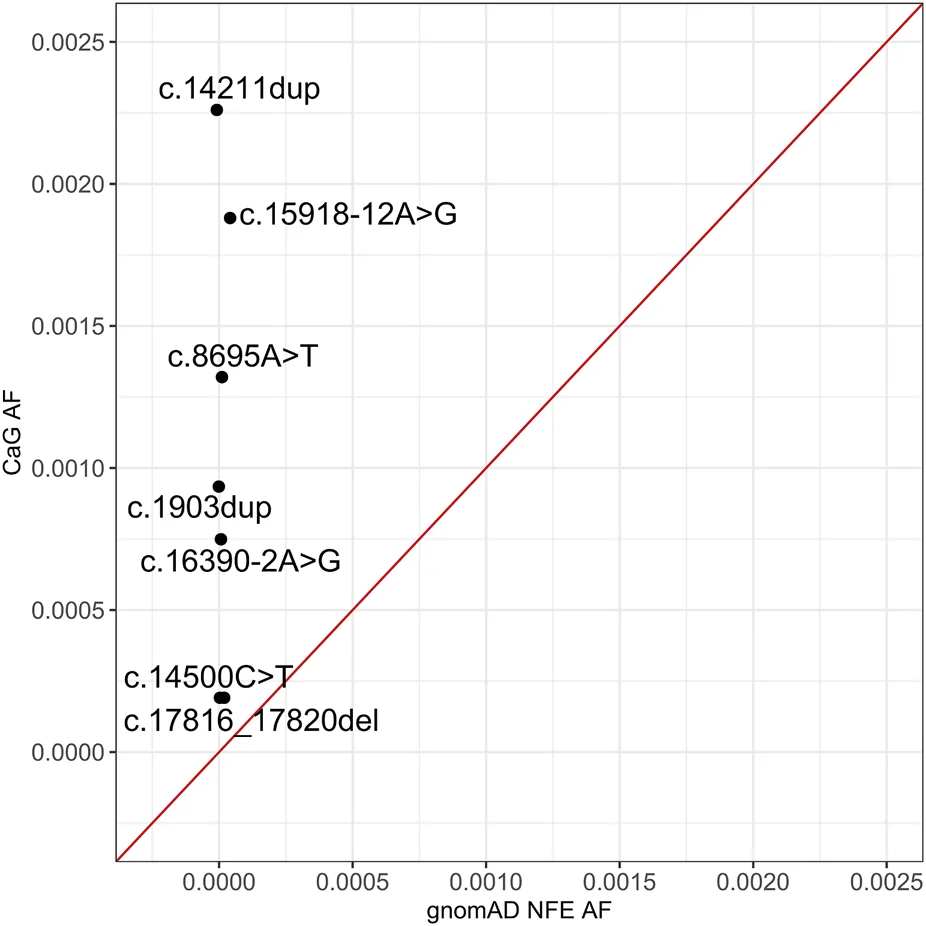
Enrichment analysis of SYNE1 variants in CaG compared to gnomAD NFE. For each identified pathogenic variant, the AF in the CaG cohort (CaG AF) is plotted against its AF in gnomAD NFE (gnomAD NFE AF). The red line denotes equal frequency in both cohorts. Variants above the line are more frequent in CaG, and those below are more frequent in gnomAD NFE.

Using the allele frequencies of all seven pathogenic *SYNE1* variants we estimated the genetic prevalence of ARCA1 in the Quebec population. The frequency of the alternate allele (q) was 0.0063 (40/6,378) giving a disease frequency (q^2^) of 3.93e-05. Assuming Hardy-Weinberg equilibrium, this corresponds to approximately 3.08 new cases of ARCA1 per year in Quebec, based on the 78,200 births in 2025, and an estimated prevalence of 3.93 per 100,000 births.

All three *SPG11* variants had higher allele frequencies in CaG than in gnomAD NFE, and all were significantly enriched in the cohort, with c.1478_1482del the most significantly enriched (Table 4; Figure 5).

**TABLE 4:** Pathogenic variants in the *SPG11* gene.

Variant	Allele count	CaG AF	CaG AF CI	gnomAD NFE AF	P-value	Odds ratio
c.6100C>T	1	1.57e-04	1.69e-05–7.33e-04	2.542e-06	0.021	61.64
c.6598A>T	4	6.27e-04	2.12e-04–1.49e-03	3.898e-05	1.58e-04	16.09
c.1478_1482del	5	7.84e-04	2.99e-04–1.72e-03	8.478e-06	1.29e-08	92.43

**FIGURE 5:**
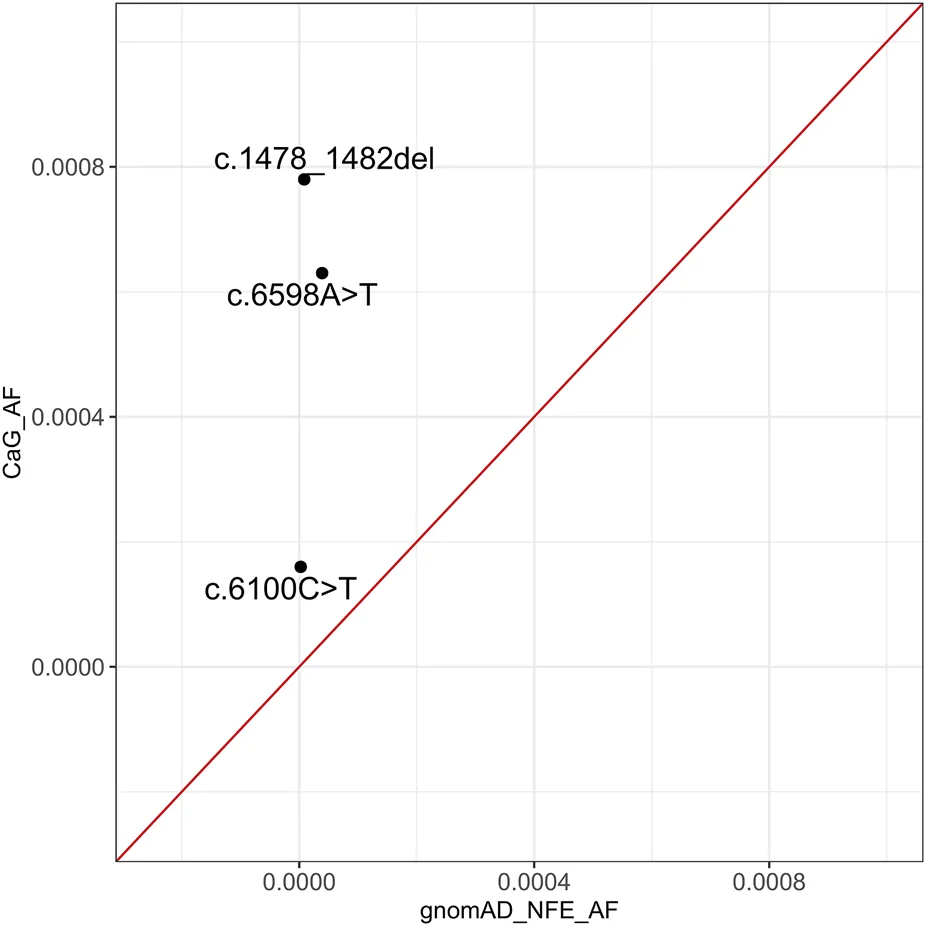
Enrichment analysis of SPG11 variants in CaG compared to gnomAD NFE. For each identified pathogenic variant, the AF in the CaG cohort (CaG AF) is plotted against its AF in gnomAD NFE (gnomAD NFE AF). The red line denotes equal frequency in both cohorts. Variants above the line are more frequent in CaG, and those below are more frequent in gnomAD NFE.

Using the allele frequencies from all three pathogenic *SPG11* variants, we estimated the genetic prevalence of HSP11 in the Quebec population. The frequency of the alternate allele (q) was 0.0016 (10/6,378), giving a disease frequency (q^2^) of 2.47e-06. Assuming Hardy-Weinberg equilibrium, this corresponds to approximately 0.19 new cases of HSP11 per year in Quebec, and an estimated prevalence of 0.25 per 100,000 births.

## 4 Discussion

This study provides the first systematic characterization of repeat expansion disorders and two neurological diseases caused by SNVs and indels in the CARTaGENE cohort, yielding population-level estimates of carrier frequency, expansion frequency, and genetic prevalence for a set of rare neurological conditions in Quebec. Because participants were drawn from the general population rather than patients affected by neurological conditions, these estimates are largely free from the diagnostic and referral biases inherent to clinical registries. Of the 19 loci analyzed, 13 harboured premutation or full-expansion alleles, indicating that pathogenic or at-risk repeat configurations are present across the general population of Quebec’s metropolitan regions. Genetic prevalence estimates for several disorders exceeded published global figures, consistent with the founder effects that have shaped the Quebec genetic landscape. Enrichment analyses of *SYNE1* and *SPG11* further identified variants at elevated frequencies relative to the NFE population in gnomAD, highlighting the province’s distinct genetic composition. Taken together, these findings have direct implications for the design of genetic screening strategies and diagnostic panels adapted to Quebec.

### 4.1 Landscape of repeat expansions in Quebec

Among the diseases expected to be enriched in the CaG cohort given their established association with the French-Canadian founder effect, *FXN* and *DMPK* variants were identified at elevated frequencies, as anticipated. The high CF of *FXN* variants is consistent with the autosomal recessive nature of FRDA, in which carriers are unaffected and therefore not excluded during recruitment of CaG participants. The recent approval of omaveloxolone as a therapy for FRDA further increases the clinical value of identifying expansion carriers early in the Quebec population (Scott et al., 2024). For DM1, the highest known prevalence globally has been reported in the SLSJ region (158 per 100,000 (Mathieu and Prévost, 2012)). Onset varies with disease severity, the congenital form is present at birth, classic DM1 has an onset of 10–30 years, and mild disease can manifest anywhere from 20 to 70 years (Bird, 1993). Notably, all three pathogenic *DMPK* alleles identified in CaG fell within the 50–150 CTG repeat range, which is associated with a milder disease phenotype, and individuals carrying such alleles may have been unaffected or unaware of their condition at the time of recruitment (Thornton, 2014).

Using PCA and IBD clustering analyses, neither the *DMPK* nor the *FXN* carriers could be assigned a specific region of origin. Some carriers grouped with an IBD cluster that is associated with the Saguenay founder effect (McClelland et al., 2026). However, only 21.5% of the cluster’s total members were recruited in Saguenay, the remainder having been recruited across other regions of the province. Membership in this cluster therefore does not imply a Saguenay-specific origin but may instead reflect shared ancestry from earlier founder populations that contributed to the settlement and subsequent expansion of populations in Saguenay as well as other regions of Quebec. Unlike DM1, FRDA is not known to be concentrated in a specific founder region such as SLSJ but occurs at high frequency across the French-Canadian population, its origin having been traced to a specific founder couple (Barbeau et al., 1984). FRDA therefore serves as a useful comparison, as the similar distributions of the *FXN* and *DMPK* carriers support the presence of *DMPK* premutation carriers outside the SLSJ region. These carriers are at risk of transmitting DM1, as instability in the repeat can lead to larger expansions. Genetic counselling programs for DM1 have been available in SLSJ since 1981, with predictive testing offered to at-risk individuals since 1988 (Mathieu and Prévost, 2012). The presence of premutation carriers across multiple regions suggests that similar screening and counselling could be extended to other regions of the province.

Before the discovery of the *FGF14*-GAA expansion as the cause of SCA27B, SCA2 was considered the predominant form of dominant inherited cerebellar ataxia in Quebec, accounting for 47% of autosomal dominant spinocerebellar ataxia (AD-SCA) diagnoses in the province (Alshimemeri et al., 2023). In the CaG cohort, the *FGF14*-GAA expansion was the most frequently detected dominant ataxia-associated expansion. Approximately one in 218 individuals carried a reduced-penetrance allele, and one in 546 carried a fully penetrant allele. The expansion was previously identified in six French-Canadian patients with unsolved late-onset cerebellar ataxia (LOCA) and in 61% of French-Canadian patients in a LOCA cohort (Pellerin et al., 2023). A parallel study of Canadian ataxia patients confirmed it as the most common cause of adult-onset dominant SCA in French-Canadian families (Cuillerier et al., 2025). Together, the concentration of *FGF14*-GAA expansions among French-Canadian ataxia patients and the high carrier frequency observed in this cohort establish SCA27B as the leading form of dominantly inherited ataxia in Quebec, with a frequency that likely reflects the founder effect. As SCA27B has only recently been described, its global prevalence is currently unknown. Our value of 55.28 per 100,000 is therefore among the first population-level genetic prevalence estimates for this disorder. The large size of the *FGF14* expansion is specifically challenging to detect accurately with short-read sequencing approaches, therefore prevalence could be overestimated. Nevertheless, the elevated frequency of SCA27B risk alleles in CaG supports the increased presence of this condition in Quebec. SCA2’s estimated genetic prevalence in CaG was consistent with current prevalence estimates, falling at the upper end of the current reported range (1–2 per 100,000 (Orphanet, 2026)) and higher than the minimum prevalence estimated in Eastern Quebec (1.06 per 100,000 (Haj Salem et al., 2021)). SCA27B and SCA2 were the only AD-SCAs for which a pathogenic expansion was detected in CaG, reinforcing that they are the two most common AD-SCAs among French Canadians. Genetic screening for ataxia should therefore prioritize both disorders, particularly SCA27B which is likely underrepresented in existing diagnostic panels as it is a recently described expansion. This is especially relevant as SCA27B patients, including individuals from Quebec, have been misdiagnosed with *CACNA1A*-related disorders because of phenotypic overlap (Indelicato et al., 2025). Although no therapy is yet approved for SCA27B, its reported responsiveness to 4-aminopyridine makes early and accurate diagnosis particularly valuable (Pellerin et al., 2024).

The *C9orf72* hexanucleotide repeat expansion was among the findings with the most marked enrichment relative to global estimates. Shortly after its discovery as a genetic cause of ALS/FTD, the expansion was shown to segregate with the disease in three French-Canadian families, and a rare variant analysis of ALS in French Canadians detected expansions in 18.6% of ALS cases and in no controls (Daoud et al., 2012; Ross et al., 2024). In the CaG cohort, ALS/FTD showed one of the highest pathogenic expansion frequencies of all conditions examined, with an estimated genetic prevalence approximately 4.6 times higher than the upper range of the estimated prevalence of ALS (5–12 per 100,000 (Gossye et al., 1993)) and 6-fold higher for FTD (9.17 per 100,000 (Urso et al., 2025)). This substantial enrichment highlights the elevated contribution of *C9orf72* expansions to neurodegeneration in the Quebec population.

Premutation alleles in *FMR1*, associated with fragile-X related disorders, were identified at similar frequencies in male and female participants (one in 248 and one in 298, respectively), and no full expansions were detected. Prior studies have reported shared founder haplotypes among *FMR1* premutation carriers in Quebec, consistent with the founder effect in the French-Canadian population, and elevated *FMR1* premutation rates in French-Canadian women (Rousseau et al., 1995). However, common premutation rates in females are not unique to Quebec, one study reported a CF of one in 201 in a large ethnically diverse population of women (Owens et al., 2018). Male premutation carriers are at a higher risk of developing FXTAS, with 30–40% affected after age 50 and penetrance increasing with age (Movaghar et al., 2019; Jacquemont et al., 2004). Approximately 8–16% of female premutation carriers develop FXTAS, and some develop fragile X-associated primary ovarian insufficiency (FXPOI) (Movaghar et al., 2019). Symptoms in female carriers tend to be milder, but the repeat is prone to expansion when transmitted from mother to child (Jacquemont et al., 2004). Penetrance correlates strongly with the number of CGG repeats, with FXTAS more common above 70 repeats (Jacquemont et al., 2004). Two of our male carriers had 75 and 79 CGG repeats, placing them at higher risk of developing FXTAS. Overall, the premutation carriers in CaG are at risk for FXTAS later in life and female carriers are additionally at risk of FXPOI and may transmit an expanded *FMR1* allele that can cause FXS in their children.

Beyond the diseases expected to be enriched through the Quebec founder effect, several findings emerged that were not anticipated from the prior literature. Spinocerebellar ataxia 17 (SCA17), caused by CAG/CAA repeat expansions in *TBP*, is predominantly reported in East Asian populations, though cases have been described in French Canadians (Ibañez et al., 2024; Alshimemeri et al., 2023). A premutation CF of one in 273 in CaG suggests the condition may be more prevalent in Quebec than previously recognized, with all alleles falling in the 41–43 repeat range. This is noteworthy given recent studies showing that intermediate *TBP* expansions (40–49 repeats) frequently co-occur with spinocerebellar ataxia 48-associated *STUB1* variants and can modify the phenotype, contributing to more severe or fully penetrant ataxia (Barbier et al., 2023; De Winter et al., 2025). However, none of the premutation carriers in CaG carried a *STUB1* variant. Intermediate-range *TBP* expansions have also been reported to result in reduced penetrance, suggesting a potential risk of reduced-penetrance SCA17 in the Quebec population (Oda et al., 2004).

Huntington’s disease like-2 (HDL2), caused by CTG expansions in *JPH3*, is observed primarily in individuals of African ancestry and is considered rare in European populations (Ibañez et al., 2024). The identification of three premutation carriers in CaG is therefore unexpected in a predominantly French-Canadian cohort. However, all four grandparents of each of these individuals were of Haitian origin. This finding underscores how immigration introduces population-specific repeat expansions into Quebec and highlights the importance of considering ancestral diversity when designing genetic screening and diagnostic programs.

SBMA, an X-linked recessive disorder caused by a CAG expansion in *AR*, had an estimated male genetic prevalence of 4.29 per 100,000 in CaG, comparable to the reported male prevalence of 3.33 per 100,000 (Teive et al., 2024; Zanovello et al., 2023). The female genetic prevalence was estimated at 0.018 per 100,000, consistent with the rarity of the disease in female carriers. However, *AR* premutation alleles in males were relatively common with a CF of one in 331. *AR* expansions have recently been reported at unexpectedly high frequencies in general populations, around 10 times more frequent than the reported disease prevalence, possibly due to underdiagnosis, reduced penetrance or variable clinical manifestations (Zanovello et al., 2023). As SBMA’s estimated genetic prevalence in Quebec remained close to its expected global frequency, the founder effect does not appear to have elevated SBMA disease in the province as it did for other REDs, unlike *FXN* and *DMPK*, which have documented founder origins in this population. The relatively high premutation CF nonetheless shows that *AR* alleles are more widely present in the population than the disease prevalence alone would suggest, consistent with this being a broader pattern at this locus rather than a Quebec-specific enrichment.

### 4.2 Prevalence of ARCA1 and HSP11

We estimated the genetic prevalence of ARCA1 and HSP11 in the Quebec population. Analysis of WGS, RNA and exome-sequencing from the CaG cohort identified seven pathogenic variants in *SYNE1* and three in *SPG11*. The allele frequencies of these variants were used to estimate prevalence and to determine whether the variants are enriched in our population.

ARCA1 was initially discovered in French Canadians, concentrated in the Beauce and Bas-Saint-Laurent regions of Quebec (Dupré et al., 2007). Although recent studies have reported heterogeneous clinical phenotypes in other countries, it remains highly prevalent in the French-Canadian population, where it is the most prevalent autosomal recessive hereditary ataxia in Eastern Quebec (Haj Salem et al., 2021; Arias et al., 2022).

The seven variants identified in *SYNE1* were classified as pathogenic or likely pathogenic in ClinVar. Six are predicted to introduce a premature stop codon, leading to loss of normal protein function, the exception being c.1903dup, identified in a patient with ataxia (Dupré et al., 2007; ClinVar, 2022; ClinVar, 2026; ClinVar, 2024). The most frequent *SYNE1* variant in CaG was c.14211dup. The second most frequent variant, c.15918–12A>G, was reported to have a higher frequency in individuals from the Beauce region than in those from urban areas, CaG genotyping and WGS data forming part of the urban comparison group in that study (Gagnon et al., 2025). Four of the variants had already been reported in French-Canadian ataxia patients in the initial description of ARCA1, but c.17816_17820del was the only variant which was not enriched significantly in the CaG cohort, although its p-value (0.052) was very close to the significance threshold (Dupré et al., 2007). A 2016 study characterized *SYNE1* variants in non-Canadian patients from Europe (Synofzik et al., 2016). Consistent with their rarity in the gnomAD NFE cohort, none of the variants we identified in CaG were among those reported, supporting an enrichment in the French-Canadian population and a potential founder effect.

HSP11 is the most prevalent autosomal recessive form of HSP, representing up to 8% of cases worldwide and the most common in Canada (Chojdak-Łukasiewicz et al., 2023). Its global prevalence is estimated at 0.34 per 100,000 (Chojdak-Łukasiewicz et al., 2023). Variants in *SPG11* are the third most common cause among Canadian HSP patients and are important predictors of disability as they are associated with progressive cognitive deficits (Chrestian et al., 2017). The c.6598A>T variant was found at higher frequency in Beauce than in the urban regions of Quebec, and all three *SPG11* variants were significantly enriched in the CaG cohort compared to the gnomAD NFE cohort, suggesting an association with the regional founder effect (Gagnon et al., 2025).

No province-wide prevalence estimates are currently available for either ARCA1 or HSP11. A single study estimated their minimum prevalence among adult hereditary ataxias and spastic paraplegias in Eastern Quebec: 2.67 per 100,000 for ARCA1 and 0.56 per 100,000 for HSP11 (Haj Salem et al., 2021). However, most of the metropolitan areas included in the CaG cohort, namely, Greater Montreal, Sherbrooke, Saguenay and Gatineau, were not part of that study. Our estimated HSP11 genetic prevalence in CaG (0.25 per 100,000) is 2.2 times lower than the Eastern Quebec minimum (0.56 per 100,000), suggesting that HSP11 may be more concentrated in Eastern Quebec than in the more western metropolitan regions sampled by CaG. By contrast, our ARCA1 estimate (3.93 per 100,000) exceeded the Eastern Quebec minimum (2.67 per 100,000), consistent with a genetic prevalence that considers unaffected and undiagnosed carriers, not only clinical cases. Because the CaG cohort does not include the Beauce region, where ARCA1 is concentrated, this genetic prevalence likely represents a conservative estimate of the Quebec-wide prevalence. These regional differences underscore the observation in the Beauce study that distinct regions of Quebec have different demographic and genetic profiles, reinforcing the importance of fine-scale genetic analyses and tailored genetic screening programs (Gagnon et al., 2025).

### 4.3 Limitations

Some limitations also offer direction for future work. First, short-read WGS cannot precisely estimate STR size for very large repeat expansions, which would be better characterized using long-read sequencing. Uncertainty in repeat size and composition can lead to misclassification of alleles relative to pathogenic or reduced-penetrance thresholds. Because our carrier frequency, expansion frequency and genetic prevalence estimates depend directly on this classification, misclassification could influence the reported values. Secondly, the limitations of short-read sequencing, combined with a cohort containing only 2,184 individuals, converge toward a potential overestimation of the resulting carrier frequency, expansion frequency and genetic prevalence estimates, which carry wide confidence intervals. To limit this potential overestimation, we used the lower bound of the confidence interval for the prevalence of dominant and X-linked disorders. Thirdly, as CaG sampled metropolitan populations, our estimates may underrepresent other founder regions such as Beauce. These estimates should therefore be confirmed in larger cohorts with sampling extended to more regions of Quebec. Finally, the prevalences reported are genetic prevalences calculated under the assumption of Hardy-Weinberg equilibrium and full penetrance, estimating the expected number of cases and not clinically observed cases. Future work could refine these estimates by incorporating disease-specific penetrance values.

## 5 Conclusion

Using sequencing data from the CaG cohort, we estimated carrier and expansion frequencies and genetic prevalences for repeat expansion disorders, as well as for the founder variants associated to ARCA1 and HSP11, in the Quebec population. Because these estimates were derived from the general population rather than clinical case registries, they avoid the diagnostic biases that may limit prevalence estimates for these rare diseases. Several disorders, including DM1, FRDA, SCA27B, and ALS/FTD, were enriched relative to global estimates, consistent with the founder effects that shape the Quebec genetic landscape, and we provide among the first population-level genetic prevalence estimates for recently described REDs such as SCA27B. Several findings were not anticipated from the prior literature, including a high frequency of SCA17 premutation alleles and the identification of HDL2 in individuals of Haitian descent. Together, these results establish a genetic profile of rare neurological diseases in the general Quebec population that can inform genetic diagnosis, disease-risk evaluation, and the design of region-adapted screening panels. They also highlight the need to implement disease registries, as many of these disorders could be underdiagnosed, are clinically heterogeneous, or have only recently been described. Extending this work to larger cohorts, additional founder regions, and long-read sequencing will further refine the genetic landscape of repeat expansions among other neurological disorders in the province.

## Data Availability

The data analyzed in this study is subject to the following licenses/restrictions: access to the CARTaGENE (CaG) genetic and phenotypic data is controlled in accordance with the consent provided by participants. Requests to access these datasets should be directed to access@cartagene.qc.ca, https://portail.cartagene.qc.ca/data-access-process.
