# Supplementary material for: Genetic prevalence of repeat expansion and other rare neurological disorders in the Quebec population
Source: Front Genet. 2026 Oct 21;17:1964104. doi: 10.3389/fgene.2026.1964104 (PMC13730467; doi:10.3389/fgene.2026.1964104)
Supplement: Supplementary file 1 [file Supplementaryfile1.docx]

Supplementary Material

# 1 Supplementary Figures and Tables

## 1.1 Supplementary Figures

**Supplementary Figure 1.** Geographic reference map of Quebec`s administrative regions. The 17 distinct administrative regions of Quebec are shown, each numbered and colored, identified by their official name. Regions relevant to this study include Saguenay-Lac-Saint-Jean (02), Bas-Saint-Laurent (01), Charlevoix within Capitale-Nationale (03) and Beauce within Chaudière-Appalaches (12). Region boundaries were obtained from the Ministère des Ressources naturelles et des Forêts du Québec (« Découpages administratifs, » Données Québec) and are reproduced under the Creative Commons Attribution 4.0 International (CC BY 4.0) license.

**Supplementary Figure 2.** Regional genetic affinity of *DMPK* and *FXN* premutation and full-mutation carriers assessed by principal component analysis. *DMPK* (A, B) and *FXN* (C, D) carriers (in black) were highlighted in black on the colored PCA of CaG participants of European descent labelled by recruitment region. The left panels (A, C) show projection onto the full regional reference, and the right panels (B, D) onto a balanced subset of ~1,000 participants per recruitment region.
